# Customer Concentration, Managerial Ability, and Corporate Performance

**DOI:** 10.3389/fpsyg.2021.814646

**Published:** 2022-01-17

**Authors:** Guanghui Jin, Qingjuan Jiang, Xiaolin Liu

**Affiliations:** School of Management, Lanzhou University, Lanzhou, China

**Keywords:** customer concentration, managerial ability, corporate performance, customer-specific investment, manager heterogeneity, supplier–customer relationship

## Abstract

We examined whether and how managerial ability affects the relationship between customer concentration and corporate performance. Based on a novel measure of managerial ability, we found that customer concentration has a significant negative effect on corporate performance, while managerial ability can mitigate this effect. The negative effect of customer concentration is only significant in the subsample of low ability and lower efficiency in asset utilization, while the moderating effect of managerial ability is significant for all levels of asset utilization efficiency and more significant for firms with a lower gross margin. The results are robust to numerous robustness tests and endogeneity concerns. Additional analysis of mechanisms shows that in addition to superior operating ability, competent managers select major customers who are more beneficial to their company and decrease the sensitivity of their research and development (R&D) investment to customers. These findings indicate that the heterogeneity of managerial ability plays an important role in the supplier–customer context when the supplier firm generally faces one or more concentrated customers.

## Introduction

While a large volume of literature has investigated the relationship between customer concentration and corporate performance, the results are mixed. Some studies have found that customer concentration enhances corporate performance (Patatoukas, 2012; Irvine et al., 2016), while others have found just the opposite that customer concentration may extract the value accrued to a supplier firm (Hui et al., 2019; Cohen and Li, 2020). The contradictory results depend on the nature of the supplier–customer relationship (Irvine et al., 2016), the own power of the supplier firms (Hui et al., 2019), or the customer type (Cohen and Li, 2020). Despite the above-mentioned influencing factors related to customer or supplier firm characteristics, managerial ability, as a more active factor, has seldom been investigated. This study examines whether and how managerial ability affects the relationship between customer concentration and corporate performance.

Drawing on one primary research theme in behavioral finance – that is, that manager heterogeneity affects various corporate decisions – we posited that managerial ability may reduce the value extraction of major customer concentration from supplier firms. Managers with a high level of ability will behave differently in the face of major customer concentration relative to low-ability managers. First, high-ability managers may intentionally choose major customers who are more beneficial to their firm; enhance the competitiveness of their product or service; or decrease customer-specific investment, reduce dependence on major customers to the maximum extent possible, and, accordingly, mitigate the value entrenchment of major customers. Second, many studies have shown that high-ability managers have superior operational skills (Choi et al., 2015), can better predict changes in the economic prospects of a firm (Baik et al., 2011), and, accordingly, improve corporate performance *via* efficient asset utilization.

To test our prediction, we used a novel measure of managerial ability developed by Demerjian et al. (2012), which is being used increasingly in accounting and finance studies, focusing on “managers’ efficiency in transforming firm resources into revenues compared to industry peers.” We mainly tested the moderating effect of managerial ability on the relationship between customer concentration and corporate performance. We found that customer concentration has a significant negative effect on corporate performance, while managerial ability can mitigate this effect.

We then used a cross-sectional test to show that the negative effect of customer concentration is only significant in the subsample of low ability and lower efficiency in asset utilization, while the moderating effect of managerial ability is significant for all levels of asset utilization efficiency and is more significant for firms with a lower gross margin that may face fiercer competition in the market. Furthermore, we found that for all firms, asset utilization efficiency increases with managerial ability. Collectively, our evidence shows that superior operational ability can effectively alleviate the negative impact of customer concentration on corporate performance.

One potential endogeneity concern is that our baseline and moderating model will suffer from an omitted variable that correlates with both customer concentration and managerial ability. The other endogeneity concern is reverse causality, in which low-performing companies have greater customer concentration and then require high-ability managers to improve performance. To alleviate these endogeneity problems, we adopted two different types of propensity score matching (PSM), the first is based on customer concentration and the second on managerial ability, and the instrumental variable approach. We adopted regional blood donation as our instrumental variable, the underlying argument being that blood donation in a region might reflect the trust level among people (Guiso et al., 2004), which would enhance trust between companies and result in more transactions. Consequently, this would positively correlate with customer concentration, while having no direct correlation with corporate performance. At the same time, senior executives in this area would pay more attention to their reputation and appraisals of outsiders of the firm, and then display superior ability. After considering the endogeneity issues, our results remain the same.

We also performed a series of robustness tests. These tests include a lag of the explanatory variables, replacement of the dependent variable with return on assets (*Roa*) and industry-adjusted return on equity, exchange of the independent variable for a customer concentration dummy, adoption of 2-year lagged customer concentration as an additional instrumental variable, exclusion of monopoly industries, further controlling whether the chief executive officer (CEO) was a “star” manager, exclusion of samples with a customer concentration of one, and retention of samples with a sales rate of the largest customer being bigger than 10%. These tests do not affect the results.

Additionally, we examined other possible mechanisms of how managerial ability may play a moderating effect. Specifically, high-ability managers may select major customers who are more beneficial to their company or increase research and development (R&D) investment to enhance the competitiveness of their product or service to reduce dependence on their major customers as far as possible. We documented that managerial ability is positively correlated with customer concentration, while high-ability managers prefer to deal with non-listed companies. We did not find any evidence to support high-ability managers spending more on R&D for their firm. However, in a much smaller listed customer sample, which could provide the research data needed, we found that customer R&D investment is positively correlated with supplier R&D investment, while managerial ability can significantly decrease the sensitivity of the R&D investment of the company to customers. From this perspective, the results show that high-ability managers always try to reduce their dependence on major customers, even when facing a larger listed company.

This study makes the following principal contributions to the literature. First, it extends literature that has examined how customer concentration affects corporate performance. After considering the research design problem, recent literature has found that customer concentration is negatively correlated with corporate performance. Some studies have further examined the different effects of customer concentration through variables related to either customer characteristics (Cohen and Li, 2020) or supplier characteristics (Hui et al., 2019). This study extends these findings by showing that managerial ability, as a more active factor, can mitigate the negative effects of major customer concentration on corporate performance.

Second, this study contributes to the literature on how an important characteristic of managers – namely, ability – affects important corporate decisions. Prior research has documented that high-ability managers are superior not only in accounting judgment and estimation (Demerjian et al., 2013; Choi et al., 2015) but also in various financial decisions (Chen et al., 2015; Andreou et al., 2017; Koester et al., 2017; Yung and Chen, 2018; Gan, 2019). We extended this line of research to the customer−supplier context to show that superior ability in large customer management is an important determinant of corporate performance.

Third, this study provides a mechanism to analyze how managerial ability mitigates the negative effect of customer concentration on corporate performance. Besides their superior operating ability, able managers do not invest more in R&D to improve their product or service but select major customers who are more beneficial to their company and decrease the sensitivity of their R&D investment to customers.

The remainder of the study is organized as follows. The section “Related Literature and Hypothesis Development” provides the literature review and develops our hypotheses. The section “Data and Model Specification” describes our sample and explains the empirical methodology. The section “Empirical Results” presents and discusses the empirical results. The section “Conclusion” concludes the study.

## Related Literature and Hypothesis Development

### The Effect of Customer Concentration on Corporate Performance

Maintaining stable relationships with major customers is crucial to corporate operating performance, and customer concentration is also an important corporate resource. Consequently, acquiring or losing a major customer is usually regarded as a significant event for any company. The relationship between customer concentration and corporate performance has been examined extensively, but the results have been inconclusive. One research branch has provided evidence that customer concentration enhances corporate performance, which we defined as the value creation effect. The effect represents a competitive advantage for companies to have a concentrated customer base. Concentrated customer relationships facilitate product cooperation and inventory management and enhance corporate performance through efficient asset utilization and expenditure reduction (Patatoukas, 2012). With a mature supplier–customer link, the key value drivers of customer-specific investment benefit supplier firms through decreased costs, increased operating efficiency, and technology sharing (Irvine et al., 2016).

Another branch of research has provided evidence that customer concentration is detrimental to corporate performance, which we defined as the value entrenchment effect. This argument focuses on the stronger bargaining position of major customers when negotiating trade contracts, on the one hand, and the role of the double-edged sword of customer-specific investment, on the other. Although customer-base concentration can help suppliers achieve effects of scale, the incremental value may be extracted by major customers. Customer-specific investment is a key value driver for suppliers, but it is usually useless outside the relationship, plunging the company into higher demand uncertainty and stickier selling, general, and administrative expenses, especially in the early stages of the supplier–customer relationship (Irvine et al., 2016), or for major customers (Cohen and Li, 2020). Supplier firms with major customer concentration may face higher risks (Dhaliwal et al., 2016), serious problems of delay, or high switching costs (Dasgupta et al., 2021). Considering sample selection, measurement, and interpretation issues, customer-base concentration is negatively correlated with corporate performance (Hui et al., 2019). As the basis of our follow-up hypothesis (H2), we put forward the following initial hypothesis:

**H1:** Customer concentration affects corporate performance negatively.

### The Moderating Effect of Managerial Ability

In the follow-up research on customer concentration and its impact, some studies have further investigated this theme from the perspective of customer types. For instance, major government customers contribute to corporate profitability, while major corporate customers act to their detriment (Cohen and Li, 2020). Despite the prior evidence, less is known about the role that managerial ability plays in the supplier–customer context.

We predict that managerial ability can mitigate the negative relationship between customer concentration and corporate performance. Our theoretical argument is mainly based on how manager heterogeneity matters to corporate decisions – a primary focus in the study of behavioral finance. According to upper echelons theory, organizational outcomes are somewhat reflections of the cognitive bases of powerful actors in the organization, that is, the CEOs or top management teams (TMTs) (Hambrick and Marson, 1984; Hambrick, 2007). Contrary to this theory, which focuses more on the observable characteristics of CEOs or TMTs, some studies in behavioral finance have suggested that certain unobservable characteristics of CEOs can significantly affect corporate decisions and outcomes, including fixed effects of managers (Bertrand and Schoar, 2003), attitudes, and personality traits (Malmendier and Tate, 2005, 2008; Hirshleifer et al., 2012; Graham et al., 2013; Hilary et al., 2016).

Top executives (especially CEOs) require both problem-solving and interpersonal abilities to make decisions and implement them in complex organizational situations (Scholefield, 1974), whose trait scores are higher in cognitive ability, especially non-cognitive ability (Adams et al., 2018), and who are also outstanding in general ability and execution skills, have more charisma and are more strategic (Kaplan et al., 2012; Kaplan and Sorensen, 2021). Since the novel measure of managerial ability was introduced by Demerjian et al. (2012), managerial ability, as an unobservable characteristic of CEOs, has been extensively studied. Existing empirical studies have shown that managerial ability can affect accounting judgments and estimations (Demerjian et al., 2013; Choi et al., 2015); information disclosure and environment (Baik et al., 2017, 2020; Abernathy et al., 2018; García-Meca and García-Sánchez, 2018; Hasan, 2018; García-Sánchez et al., 2020); financial decision-making (Chen et al., 2015; Andreou et al., 2017; Koester et al., 2017; Yung and Chen, 2018; Gan, 2019; Dong and Doukas, 2021); and accounting, capital markets, and corporate social responsibility (CSR) performance (Demerjian et al., 2012; Mishra, 2014; Bonsall et al., 2017; Cheung et al., 2017; Yuan et al., 2019). Although the results have been inconclusive, evidence from numerous empirical studies has demonstrated that managerial ability does play a crucial role in the operation and fundamentals of a firm.

We posited that high-ability managers behave differently, compared with low-ability managers, when facing major and concentrated customers. First, able managers are more knowledgeable about their business and major customers (Demerjian et al., 2012). They may purposefully choose and maintain major customers who are more beneficial to the company and enhance corporate performance *via* business development.

Even when facing major customers with strong negotiation power, high-ability managers can improve unfavorable situations through efficient operational decisions and implementation (Choi et al., 2015). They can better anticipate changes in the economic environment of their firms (Baik et al., 2011) and thereby forecast demand more accurately, purchase quality items, manage inventory and receivables efficiently, and improve corporate performance through improved resource deployment.

Second, an important reason for the value entrenchment effect of customer concentration concerns customer-specific investment. This is useless to outside customers, causing suppliers to face serious delay problems, relying more on major customers and thus losing the negotiating power of price and becoming locked into a long-term relationship, with negative consequences to the firm. To obtain bargaining power in bilateral deals, high-ability managers may make more innovative investments to improve the competitiveness of their products or services (Chen et al., 2015), especially under the booming development of the Internet of things and big data technologies (Kovacova et al., 2020; Kovacova and Lewis, 2021). Otherwise, they may deliberately reduce relationship-specific investments to avoid the value entrenchment of major customers by lowering prices. High-ability managers are more likely to make long-term commitments, such as CSR investment (Yuan et al., 2019). Furthermore, the reputation capital of high-ability managers fosters trust in the deal with major customers and can both substitute the relationship-specific investment and ameliorate disadvantages in the bilateral relationship.

Taken together, the managerial ability has a substantial impact on the relationship of a firm with major and concentrated customers. Accordingly, we put forward the following hypothesis:

**H2:** The negative effect of customer concentration on corporate performance is weaker for firms with high-ability managers.

## Data and Model Specification

### Sample and Data

This study utilizes Chinese A-share companies listed on the Shanghai and Shenzhen Stock Exchanges from 2010 to 2019 as a research sample. The decision on timeframe was made because the disclosure of top-five customers of a firm was voluntary before 2009; hence, to reduce bias in the sample selection caused by voluntary disclosure of a firm, we opted to use data after 2009. Furthermore, we excluded the financial and insurance industries, “ST” and “*ST”, as well as firms with missing financial data. Finally, 19,953 firm-year observations were obtained. The data concerning customer concentration were derived from the China National Research Data Service (CNRDS) database, the data for managerial ability were calculated using the two-step methodology developed by Demerjian et al. (2012), and the other main financial data were obtained from the China Stock Market and Accounting Research (CSMAR), CNRDS, and Wind databases. To eliminate the influence of extreme values, we winsorized all continuous variables at the 1st and 99th percentiles. Stata 15.0 was used for data processing and analysis.

### Variable Measurement

#### Customer Concentration

Most listed companies in our sample disclosed the total revenue of their top-five customers, while some companies only disclosed the revenue of each of the top-five customers separately. To reduce the problem of sample self-selection and to avoid missing a large number of samples, we measured customer concentration (*CC*) as the ratio of top-five customer sales divided by the total annual sales of the firm.

#### Managerial Ability

We used the two-step methodology developed by Demerjian et al. (2012) to calculate managerial capability. First, the data envelopment analysis (DEA) was used to calculate the full efficiency value of the sample by industry and year, and we then standardized the calculated values. Specifically, we assigned the most effective sample in the industry to one and calculated the relative efficiency value of other companies.


(1) $$M\,\text{ax}\,F\,e=\frac{S\,a\,l\,e\,s}{\varphi_{1}\,C\,o\,g\,s+\varphi_{2}\,P\,p\,e+\varphi_{3}\,R\&d+\varphi_{4}\,G\,w+\varphi_{5}\,S\,g\&a+\varphi_{6}\,I\,n\,\tan\,g}$$

In model (1), *Sales* is the revenue of firm as the output variable, and the input variables are operating costs (*Cogs*), net fixed assets (*Ppe*), R&D expenditures (*R*&*d*), goodwill (*Gw*), firm sales, and management expenses (*Sg*&*a*), and net intangible assets (*Intang*); all can be obtained from the publicly disclosed financial reports of the firm.

The efficiency value generated by the above DEA model can be attributed to the firm-specific factors or management characteristics. Therefore, in the second step, we used Tobit regression to remove the efficiency value derived from the firm-specific characteristics. Specifically, we chose some variables to represent the firm-specific characteristics, including firm size (*Size*), market share (*Ms*), free cash flow (*Fcf*), firm age (*Age*), firm diversification (*Bsc*), and firm internationalization (*Oos*). We then regressed these characteristics to the efficiency value derived from model (1), shown as the model (2).


(2) $$F\,e=\phi_{0}+\phi_{1}\,S\,i\,z\,e+\phi_{2}\,M\,s+\phi_{3}\,F\,c\,f+\phi_{4}\,A\,g\,e+\phi_{5}\,B\,s\,c+\phi_{6}\,O\,o\,s+\sum Y\,e\,a\,r+\mu$$

We posited that the remaining efficiency cannot be explained by the firm-specific characteristics that can be accrued to managerial ability. Thus, we used the regression residuals of model (2) as our proxy of managerial ability.

#### Corporate Performance

We used the return on equity (*Roe*) to measure corporate performance, which reflects the comprehensive operating outcome of the firm, corresponding to the residual efficiency value accrued to managerial ability. We also used the *Roa* as our additional proxy for corporate performance for robustness testing.

#### Control Variables

We controlled some other variables that have an impact on corporate performance, including firm size (*Size*), growth capability (*Grow*), leverage (*Lev*), ownership concentration (*First*), firm age (*Age*), the ratio of fixed assets (*Tangible*), independent directors (*Indd*), firm nature (*State*), board size (*Board*), book-to-market ratio (*Mb*), executive compensation (*Pay*), degree of separation of two rights (*Separation*), and cash flow (*Cf*). Detailed definitions of the variables are shown in Appendix.

### Model Specification

We estimated model (3) to examine the relationship between customer concentration and corporate performance and model (4) to examine the moderating effect of managerial ability.


(3) $$R\,o\,e_{i,t}=\alpha_{0}+\alpha_{1}\,C\,C_{i,t}+\alpha_{2}\,C\,o\,n\,t\,r\,o\,l\,s_{i,t}+\sum Y\,e\,a\,r+\sum I\,n\,d+\nu_{i,t}$$

(4) $$R\,o\,e_{i,t}=\beta_{0}+\beta_{1}\,C\,C_{i,t}+\beta_{2}\,A\,b\,i\,l\,i\,t\,y_{i,t}+\beta_{3}\,C\,C_{i,t}*A\,b\,i\,l\,i\,t\,y_{i,t}+\beta_{4}\,C\,o\,n\,t\,r\,o\,l\,s_{i,t}+\sum Y\,e\,a\,r+\sum I\,n\,d+\varsigma_{i,t}$$

Here, *i* represents the firm and *t* represents the year. As described above, *Roe* is corporate performance. The explanatory variable *CC* is the sales proportion of the top-five customers. *Ability* is managerial ability. *Controls* are all the control variables mentioned in the previous section. Year and industry fixed effects are included in the regressions to control for any unobserved trends and industrial characteristics. Following Dhaliwal et al. (2016), due to the limited within-firm variation of the variable of customer concentration, we did not include the fixed effects of firms in our model. The standard errors were adjusted for heteroskedasticity and clustered at the firm level. We expected α_1_ in model (3) to be significantly negative following H1 and β_3_ in model (4) to be significantly positive according to H2.

### Summary Statistics

Table 1 provides the descriptive statistics of our main variables. The mean value of *CC* is 0.303, implying that the customer concentration of listed companies in China is at a high level, while the standard deviation is 0.216, smaller than the mean, showing that there is less variation of *CC*. The average *Roe* is 6.3%, which means that, on average, the corporate performance is adequate, but the variation therein is great. The average value of managerial ability (*Ability*) is −0.005, whereas its standard deviation is 0.154, indicating that the heterogeneity of managerial ability is very large. Hence, it is necessary to study the heterogeneous influence of managerial ability in a relatively stable supplier–customer context.

**TABLE 1:** Descriptive statistics.

Variable	Observations	Mean	SD	Min.	Max.
*Roe*	19,953	0.063	0.125	−0.777	0.350
*CC*	19,953	0.303	0.216	0.000	1.000
*Ability*	19,953	−0.005	0.154	−0.674	0.458
*Size*	19,953	22.11	1.275	19.33	26.06
*Grow*	19,953	0.426	1.187	−0.713	9.096
*Lev*	19,953	0.416	0.208	0.050	0.980
*First*	19,953	0.351	0.147	0.087	0.750
*Age*	19,953	2.028	0.873	0.000	3.296
*Tangible*	19,953	0.212	0.156	0.002	0.720
*Indd*	19,953	0.394	0.093	0.143	0.667
*State*	19,953	0.355	0.479	0.000	1.000
*Board*	19,953	2.131	0.195	1.609	2.708
*Mb*	19,953	0.614	0.241	0.115	1.147
*Pay*	19,953	15.27	0.715	13.07	17.22
*Separation*	19,953	0.046	0.074	0.000	0.284
*Cf*	19,953	0.044	0.070	−0.187	0.257

An average firm in our sample has a size of 22.11, operating income growth rate of 42.6%, leverage of 41.6%, firm age of 2.028 years, the proportion of fixed assets of 21.2%, a book-to-market ratio of 0.614, and cash flow of 0.044. In addition, the average shareholding proportion of the largest shareholder is 35.1%, indicating that it is a controlling shareholder in our sample. State-owned companies make up 35.5% of our sample, independent directors amount to 39.4% in an average board size of 2.131, the average degree of separation of the rights of control and cash flow is 0.046, and the monetary compensation of senior executives in the sample firms is 15.27, which is about 5.7 million a year on average.

## Empirical Results

### Baseline Results

In Table 2, we explored the main effect of customer concentration on corporate performance and report the empirical results of model (3). The first column of Table 2 contains the univariate regression result. In the second column of Table 2, we included a full set of control variables, consistent with H1, the coefficient of *CC* (β = −0.0113, *t* = −2.08, *p* < 0.05) is significantly negative, which shows that firms with a higher customer concentration have worse performance. Specifically, an increase in the revenue share of the top-five customers by one standard deviation will lead to a decline in the corporate performance of 3.87%.

**TABLE 2:** Main and moderating effects.

	(1)	(2)	(3)	(4)
	*Roe*	*Roe*	*Roe*	*Roe*
*CC*	−0.0340***	−0.0113**	−0.0445***	−0.0205***
	(−5.24)	(−2.08)	(−6.89)	(−3.83)
*Ability*			0.2098***	0.1662***
			(20.78)	(20.64)
*CC* × *Ability*			0.1257***	0.1136***
			(3.20)	(3.38)
*Size*		0.0253***		0.0235***
		(15.99)		(14.91)
*Grow*		0.0080***		0.0079***
		(8.73)		(8.91)
*Lev*		−0.1214***		−0.1230***
		(−12.41)		(−12.84)
*First*		0.0542***		0.0485***
		(7.18)		(6.40)
*Age*		−0.0180***		−0.0172***
		(−12.52)		(−12.08)
*Tangible*		−0.0988***		−0.0760***
		(−11.54)		(−8.92)
*Indd*		0.0388***		0.0384***
		(3.47)		(3.49)
*State*		−0.0005		−0.0004
		(−0.17)		(−0.14)
*Board*		0.0093		0.0123*
		(1.42)		(1.92)
*Mb*		−0.0865***		−0.0802***
		(−14.65)		(−13.61)
*Pay*		0.0183***		0.0207***
		(9.51)		(10.84)
*Separation*		0.0586***		0.0388***
		(4.26)		(2.82)
*Cf*		0.4234***		0.3777***
		(22.47)		(21.44)
*Constant*	0.0653***	−0.6894***	0.0560***	−0.7050***
	(4.10)	(−20.37)	(3.70)	(−21.24)
Year FE	Yes	Yes	Yes	Yes
Ind FE	Yes	Yes	Yes	Yes
Observations	19,953	19,953	19,953	19,953
Adj. *R*^2^	0.016	0.213	0.076	0.249

We tested the main and moderating effect in this table. The t-statistics are displayed in parentheses. The symbols ***, **, and * indicate significance at the 1, 5, and 10% levels, respectively.

The results for the control variables are relatively consistent with those of previous literature: Corporate performance is positively associated with firm size, growth capability, ownership concentration, independent directors, executive compensation, and cash flow and is negatively associated with firm leverage and book-to-market ratio.

Next, we tested the moderating effect of managerial ability and added an interaction term (*CC* × *Ability*) to the regression. To avoid any multicollinearity problems, we centralized *CC* and *Ability* of the interaction terms *CC* × *Ability*, and the results are shown in the last two columns in Table 2. In columns (3) and (4), the coefficients of *CC* are still significantly negative (β = −0.0445, *t* = −6.89, *p* < 0.01 without control variables; β = −0.0205, *t* = −3.83, *p* < 0.01 with control variables). However, we found significant and positive coefficients on *CC* × *Ability* (β = 0.1257, *t* = 3.20, *p* < 0.01 without control variables; β = 0.1136, *t* = 3.38, *p* < 0.01 with control variables). The significantly positive sign of the interaction variable suggests that managerial ability can mitigate the value entrenchment effect of major customers, consistent with H2.

### Cross-Sectional Heterogeneity Tests

If the heterogeneity of managerial ability plays an important role in the context of the supplier–customer relationship, then we would expect that the value entrenchment effect of customer concentration and the moderating effect of managerial ability would change with the ability of managers. According to our argument, we anticipate that the higher the managerial ability, the lower the value entrenchment effect of customer concentration, and the more significant the moderating effect of managerial ability. The results of the cross-section tests are shown in Table 3.

**TABLE 3:** Cross-sectional heterogeneity tests.

	(1)	(2)	(3)	(4)	(5)	(6)	(7)	(8)
	*Roe*		*Roe*		*Roe*		*Roe*	
	*Low ability*	*High ability*	*Low Tat*	*High Tat*	*Low Tat*	*High Tat*	*Low Gm*	*High Gm*
*CC*	−0.0295***	−0.0056	−0.0130**	0.0015	−0.0198***	−0.0119**	−0.0243***	−0.0065***
	(−4.78)	(−1.15)	(−2.34)	(0.27)	(−3.66)	(−2.08)	(−3.54)	(−2.85)
*Ability*					0.1875***	0.1136***	0.1947***	0.1123***
					(24.12)	(13.70)	(20.29)	(34.03)
*CC* × *Ability*					0.0646**	0.1376***	0.1357***	0.0413***
					(2.24)	(3.77)	(3.32)	(3.22)
*Size*	0.0254***	0.0227***	0.0300***	0.0244***	0.0268***	0.0230***	0.0169***	0.0099***
	(14.69)	(15.49)	(17.84)	(16.13)	(16.38)	(15.31)	(8.47)	(14.75)
*Grow*	0.0083***	0.0076***	0.0087***	0.0097***	0.0079***	0.0097***	0.0110***	0.0014***
	(7.20)	(8.72)	(10.00)	(7.49)	(9.40)	(7.56)	(8.20)	(3.69)
*Lev*	−0.1828***	−0.0675***	−0.1549***	−0.1261***	−0.1467***	−0.1252***	−0.1895***	0.1200***
	(−24.51)	(−10.56)	(−21.95)	(−17.65)	(−21.37)	(−17.69)	(−22.55)	(36.17)
*First*	0.0564***	0.0484***	0.0533***	0.0311***	0.0484***	0.0296***	0.0501***	0.0305***
	(6.37)	(6.22)	(6.07)	(3.86)	(5.67)	(3.71)	(4.82)	(8.98)
*Age*	−0.0177***	−0.0167***	−0.0225***	−0.0144***	−0.0194***	−0.0151***	−0.0168***	−0.0064***
	(−9.25)	(−10.91)	(−12.42)	(−8.87)	(−11.02)	(−9.36)	(−7.33)	(−9.41)
*Tangible*	−0.1006***	−0.0734***	−0.0799***	−0.1214***	−0.0534***	−0.1062***	−0.1006***	−0.0189***
	(−10.35)	(−9.32)	(−8.93)	(−14.27)	(−6.10)	(−12.49)	(−9.93)	(−4.85)
*Indd*	0.0527***	0.0219*	0.0465***	0.0288**	0.0448***	0.0292**	0.0546***	0.0072
	(3.70)	(1.67)	(3.27)	(2.16)	(3.24)	(2.21)	(3.24)	(1.26)
*State*	−0.0004	−0.0025	−0.0025	−0.0020	−0.0018	−0.0016	0.0146***	−0.0017
	(−0.13)	(−0.92)	(−0.80)	(−0.72)	(−0.59)	(−0.56)	(4.16)	(−1.33)
*Board*	0.0235***	−0.0066	0.0055	0.0140**	0.0090	0.0151**	0.0213***	−0.0002
	(3.50)	(−1.05)	(0.82)	(2.26)	(1.37)	(2.45)	(2.73)	(−0.06)
*Mb*	−0.0516***	−0.1174***	−0.0721***	−0.0965***	−0.0602***	−0.0945***	0.0012	−0.0998***
	(−7.11)	(−18.51)	(−10.10)	(−14.82)	(−8.66)	(−14.66)	(0.13)	(−35.74)
*Pay*	0.0197***	0.0196***	0.0107***	0.0183***	0.0136***	0.0211***	0.0215***	0.0100***
	(9.04)	(10.48)	(4.94)	(9.54)	(6.47)	(11.08)	(8.74)	(11.78)
*Separation*	0.0706***	0.0239*	0.0325*	0.0655***	0.0206	0.0515***	0.0707***	0.0015
	(4.28)	(1.65)	(1.90)	(4.60)	(1.24)	(3.64)	(3.82)	(0.23)
*Cf*	0.4775***	0.3418***	0.3300***	0.4272***	0.2645***	0.4186***	0.3280***	0.3001***
	(23.51)	(22.47)	(17.37)	(25.98)	(14.18)	(25.68)	(14.82)	(41.04)
*Constant*	−0.7913***	−0.6032***	−0.6669***	−0.6663***	−0.6683***	−0.6905***	−0.6354***	−0.2404***
	(−21.95)	(−19.11)	(−18.83)	(−20.75)	(−19.39)	(−21.66)	(−15.43)	(−16.22)
Year FE	Yes	Yes	Yes	Yes	Yes	Yes	Yes	Yes
Ind FE	Yes	Yes	Yes	Yes	Yes	Yes	Yes	Yes
Observations	9977	9976	9981	9972	9981	9972	9977	9976
Adj. *R*^2^	0.234	0.206	0.201	0.237	0.247	0.251	0.188	0.473
*p*-Values	χ^2^ (1) = 7.67***		χ^2^ (1) = 3.02*		χ^2^ (1) = 1.82		χ^2^ (1) = 3.51*	

We tested the cross-sectional variations between the main effect and the moderating effect in this table; “p-values” was used to test the coefficient equality between two subsamples. The t-statistics are displayed in parentheses. The symbols ***, **, and * indicate significance at the 1, 5, and 10% levels, respectively.

To test our prediction, we first divided the sample into two groups according to managerial ability, namely, the high-ability group (*high ability*) and the low-ability group (*low ability*). The first two columns in Table 3 demonstrate that the negative relationship between *CC* and *Roe* is significant only in the group with the low ability (β = −0.0295, *t* = −4.78, *p* < 0.01), and the coefficients are significantly different between the two groups.^1^

Prior studies have shown that able managers are superior in terms of asset deployment (Baik et al., 2011; Choi et al., 2015), such as inventory management, collecting receivables, and so on. Therefore, we divided our sample into two groups based on the proxies of operational efficiency, including total asset turnover (*Tat*), accounts receivable turnover (*Art*), and inventory turnover (*It*), which equal operating income divided by total assets, accounts receivable, and inventory, respectively. Columns (3) and (4) show that the negative effect of customer concentration is only significant in the *Low Tat* subsample and is not significant in the *High Tat* subsample, while the coefficients are significantly different between the two groups. In columns (5) and (6), it is noted that the moderating effect is significant for both groups and the differences of the coefficients are insignificant between *High Tat* and *Low Tat* subsamples, which indicates that managerial ability can generally alleviate the value entrenchment of major customers in both types of company.^2^

To the extent that companies with low gross margins may face fiercer competition in the market or experience disadvantages in their negotiation positions when dealing with major customers, the role of managerial ability may be more prominent. We further used gross margin, calculated by sales revenue minus the cost of goods sold and then divided by revenue, to divide the sample into two groups: a high-margin group (*High Gm*) and a low-margin group (*Low Gm*). The results in columns (7) and (8) show that the moderating effect is significant for both subsamples, but the coefficient of the interaction term *CC* × *Ability* is significantly larger in the *Low Gm* group. Collectively, the cross-section tests further support H1 and H2.

Finally, our argument is based on the assumption that the moderation effect of managerial ability is due to the superior operational ability of the manager. Therefore, we tested whether managerial ability indeed improves corporate operating performance. The results are shown in Table 4. This shows that managerial ability is significantly positively related with all three proxies of corporate operating performance, total asset turnover (*Tat*), accounts receivable turnover (*Art*), and inventory turnover (*It*), indicating that the higher the managerial ability, the better the corporate operating performance. Collectively, we found consistent evidence that managerial ability can improve corporate performance directly or alleviate the negative impact of customer concentration on corporate performance indirectly.

**TABLE 4:** Managerial ability and asset utilization efficiency.

	(1)	(2)	(3)
	***Tat***	***Art***	***It***
*Ability*	0.9074***	2.1854***	1.0280***
	(12.20)	(15.78)	(8.72)
*Size*	−0.0482***	0.1142***	0.0189
	(−4.89)	(4.28)	(0.78)
*Grow*	−0.0312***	−0.0182	−0.0841***
	(−8.37)	(−1.46)	(−7.44)
*Lev*	0.4812***	−0.2209**	−0.0900
	(9.51)	(−2.02)	(−0.85)
*First*	0.2680***	0.6796***	0.1342
	(4.99)	(4.80)	(1.03)
*Age*	0.0237**	0.1505***	−0.0450*
	(2.08)	(5.46)	(−1.73)
*Tangible*	0.0500	2.1456***	1.8384***
	(0.91)	(13.78)	(13.76)
*Indd*	−0.0082	−0.0351	−0.2707**
	(−0.14)	(−0.25)	(−2.02)
*State*	0.0181	0.1748***	0.0906*
	(0.83)	(3.23)	(1.91)
*Board*	0.0195	0.0283	−0.0817
	(0.41)	(0.30)	(−0.94)
*Mb*	0.0332	−0.0465	0.1651*
	(0.85)	(−0.45)	(1.80)
*Pay*	0.0913***	0.0492	0.1021***
	(6.89)	(1.56)	(3.53)
*Separation*	0.0999	0.2512	0.1355
	(0.99)	(0.95)	(0.61)
*Cf*	0.5407***	2.9357***	1.2996***
	(5.64)	(12.62)	(6.93)
*Constant*	−0.1081	−1.3850***	−1.4033***
	(−0.61)	(−2.58)	(−2.87)
Year FE	Yes	Yes	Yes
Ind FE	Yes	Yes	Yes
Observations	19,953	19,953	19,953
Adj. *R*^2^	0.262	0.363	0.343

We tested the impact of managerial ability on asset utilization efficiency in this table. The t-statistics are displayed in parentheses. The symbols ***, **, and * indicate significance at the 1, 5, and 10% levels, respectively.

### Endogeneity Tests

One potential endogeneity concern was that our baseline and moderating model would suffer from an omitted variable, which is correlated with both customer concentration and managerial ability. The other endogeneity concern was reverse causality, in which companies with lower performance would have more concentrated customers and then require high-ability managers to improve their performance. To alleviate the above-mentioned endogeneity problems, we conducted several endogeneity tests, as reported in this section.

#### Propensity Score Matching Tests

We first matched the samples with customer concentration. Specifically, we defined samples with a high customer concentration as a treatment group if the leading customer accounted for more than 10% of the sales of the firm and assigned other samples to the control group. Next, we conducted Logit regression to calculate the probability that the sales ratio of the leading customer was greater than 10% based on all control variables. After calculating the propensity score of each observation, a 1:1 nearest neighbor match was performed according to the result, to generate a matched control group. The procedure yielded a matched sample consisting of 16,769 observations. There was no significant difference in all the control variables, therefore, the matching was effective. Column (1) in panel A of Table 5 reports the results based on PSM samples. Consistent with our main findings, customer concentration is negatively correlated with corporate performance.

**TABLE 5:** Endogeneity tests.

	(1)	(2)
	*Roe*	*Roe*
**Panel A: Propensity score matching**		
*CC*	−0.0104*	−0.0199***
	(−1.84)	(−3.42)
*Ability*		0.1478***
		(17.22)
*CC* × *Ability*		0.1038***
		(2.94)
*Constant*	−0.6860***	−0.6848***
	(−18.80)	(−19.07)
Controls	Yes	Yes
Year FE	Yes	Yes
Ind FE	Yes	Yes
Observations	16,769	15,392
Adj. *R*^2^	0.208	0.238
**Panel B: An instrumental variable approach**		
*CC*	−0.8297***	−0.5773***
	(−2.88)	(−2.86)
*Ability*		0.2118***
		(11.14)
*CC* × *Ability*		0.2433**
		(2.28)
*Constant*	0.1921	−0.1107
	(0.61)	(−0.51)
Controls	Yes	Yes
Year FE	Yes	Yes
Ind FE	Yes	Yes
Under identification test	χ^2^ (1) = 11.304***	χ^2^ (1) = 13.830***
Weak identification test	*F*-statistic = 11.915	*F*-statistic = 14.512
Observations	19,872	19,872

We conducted several endogeneity tests in this table, including two different kinds of propensity score matching and an instrumental variable. In panel B, the decrease in observations is due to several companies located overseas or undisclosed, “under identification test” and “weak identification test” are used to test the validity of instrumental variables. We did not report R^2^ because it is not of practical significance. The t-statistics (Z-statistics) are displayed in parentheses. The symbols ***, **, and * indicate significance at the 1, 5, and 10% levels, respectively.

In addition, we matched the high-ability and low-ability samples. Based on the median of managerial ability, we identified the treatment group with high ability. Taking *CC* and all controls as matching variables, using the same method to select the control group, 15,392 matched samples were finally obtained. We continued to observe that managerial ability alleviates the deteriorating effect of customer concentration on corporate performance in column (2) in panel A of Table 5.

#### An Instrumental Variable Approach

We also employed an instrumental variable approach to further ameliorate endogeneity bias and choose regional blood donation as our instrumental variable.^3^ Blood donation in a region may reflect the trust level among people (Guiso et al., 2004), which will enhance trust between companies and result in more transactions. This will then positively correlate with customer concentration while having no direct correlation with corporate performance. At the same time, senior executives in this area will pay more attention to their reputation and appraisals outside of the firm, and then display superior ability. Regional blood donation (*Blood*) was measured by dividing the number of blood donations by the permanent population in a region. Panel B of Table 5 reports the second-stage results. The results are consistent with the previous conclusions.

### Robustness Tests

In this section, we designed additional tests to further examine our hypotheses and conducted a battery of sensitivity tests to establish the robustness of our findings. The results are shown in Table 6.

**TABLE 6:** Robustness tests.

	Lag the explanatory variables		Dependent variable measured by *Roa*		Excluding monopoly industries		Adding control variable *Star*	
	F.Roe	F.Roe	Roa	Roa	Roe	Roe	Roe	Roe
*CC*	−0.0288***	−0.0341***	−0.0084***	−0.0142***	−0.0134**	−0.0209***	−0.0109**	−0.0202***
	(−4.53)	(−5.45)	(−2.95)	(−5.20)	(−2.34)	(−3.69)	(−2.03)	(−3.78)
*Ability*		0.0837***		0.1122***		0.1702***		0.1654***
		(10.01)		(27.34)		(20.21)		(20.59)
*CC* × *Ability*		0.1066***		0.0501***		0.1525***		0.1140***
		(3.06)		(2.91)		(4.21)		(3.40)
*Star*							0.0443***	0.0397***
							(8.50)	(7.66)
*Constant*	−0.4911***	−0.4997***	−0.3040***	−0.3157***	−0.6895***	−0.7002***	−0.6715***	−0.6888***
	(−12.75)	(−13.20)	(−18.03)	(−20.12)	(−19.50)	(−20.31)	(−19.98)	(−20.90)
Controls	Yes	Yes	Yes	Yes	Yes	Yes	Yes	Yes
Year FE	Yes	Yes	Yes	Yes	Yes	Yes	Yes	Yes
Ind FE	Yes	Yes	Yes	Yes	Yes	Yes	Yes	Yes
Observations	16,953	16,953	19,953	19,953	19,031	19,031	19,953	19,953
Adj. *R*^2^	0.147	0.156	0.319	0.386	0.211	0.249	0.214	0.250

We performed robustness tests in this table, including lagging the explanatory variables, replacing the dependent variable with Roa, excluding monopoly industries, and adding the control variable Star. The t-statistics are displayed in parentheses. The symbols ***, **, and * indicate significance at the 1, 5%, and 10% levels, respectively.

First, we lagged the explanatory variables to avoid reverse causality. In the first two columns of Table 6, the results show that the relationship between customer concentration, managerial ability, and corporate performance is robust. Second, we changed the measures of corporate performance with *Roa*. The second two columns of Table 6 show that the results are qualitatively unchanged.^4^ Third, the corporate performance of monopolistic industries may weakly correlate with customer concentration and managerial ability, which may affect our results. By excluding samples of monopolistic industries, the conclusions are in line with our hypotheses, as shown in the third two columns of Table 6.^5^ Finally, we further controlled for whether the CEO of the company was a star CEO, which was defined as the ‘‘best CEO of a listed company in China’’ selected by Forbes China.^6^ This was assigned a value of 1, and 0 otherwise. In the last two columns of Table 6, it is noted that after adding the control variable of *Star*, the results remain the same as our baseline results, which indicates that when considering some other individual characteristics of managers, their ability still has an incremental explanatory power.

Overall, these additional tests reinforce our key findings that corporate performance decreases with customer concentration, but the managerial ability can mitigate this effect.

### Analysis of Mechanisms

In addition to superior operational ability, we examined other possible mechanisms by which managerial ability can alleviate the deteriorating effect of customer concentration on corporate performance. One possible mechanism may be that high-ability managers select major customers who are more beneficial to their company. To test this conjecture, we analyzed detailed information about the types of customers disclosed by the company. Generally, a listed company has stronger bargaining power in bilateral deals between suppliers and customers. In this case, able managers may intentionally deal with non-listed companies. To begin, we tested the relationship between managerial ability and customer concentration. Then, we used the sales ratio of listed and non-listed companies among the top-five customers as a measure of the concentration of listed customers (*CC_list*) and non-listed customers (*CC_nonlist*) and examined the effect of managerial ability on these two concentrations. Due to the absence of detailed information about the type of customers, the sample is smaller than the baseline regression. The results are shown in Table 7. Column (1) of Table 7 shows that managerial ability is positively correlated with customer concentration, indicating that the greater the competency of managers, the greater the concentration of major customers. However, in columns (2) and (3) in Table 7, it is noted that managerial ability (*Ability*) is only significantly positively related to the concentration of non-listed customers (*CC_nonlist*), which confirms our conjecture that high-ability managers prefer to deal with non-listed companies, who may be more beneficial to their company rather than listed companies.

**TABLE 7:** The mechanism of customer selection.

	(1)	(2)	(3)
	*CC*	*CC_list*	*CC_nonlist*
*Ability*	0.0863***	0.0035	0.0611**
	(3.88)	(0.32)	(2.22)
*Constant*	1.0680***	0.0603	0.9926***
	(11.63)	(1.62)	(9.60)
Controls	Yes	Yes	Yes
Year FE	Yes	Yes	Yes
Ind FE	Yes	Yes	Yes
Observations	19,953	10,447	10,447
Adj. *R*^2^	0.143	0.036	0.161

We tested the customer selection mechanism of managerial ability in this table, using a sample of 10,447 observations that disclosed customer information in the last two columns. The t-statistics are displayed in parentheses. The symbols ***, **, and * indicate significance at the 1, 5, and 10% levels, respectively.

The other possible mechanism may be that able managers would increase R&D investment to enhance the competitiveness of their product or service (Chen et al., 2015) and reduce dependence on their major customers to the greatest extent possible. To test this prediction, we first investigated whether able managers invested more in R&D, but the results do not support our prediction.

The co-movement of R&D investment between customers and suppliers may indicate a higher degree of customer-customized investment, which offers less value outside the relationships. The specific investment in customer relationships may push supplier firms into a more dangerous situation, so we further analyzed whether managerial ability could influence the R&D co-movement between customers and suppliers. We postulated that high-ability managers are more likely to be aware of the disadvantages of customer relationship-specific investment and reduce dependence on it as much as possible. We examined whether the R&D investment of supplier firms was sensitive to customers and whether managerial ability can mitigate this sensitivity. The regression models are shown in models (5) and (6).


(5) $$S\,u\,p\,\text{\_}\,R\&d_{i,t}=\lambda_{0}+\lambda_{1}\,C\,u\,s\,\_\,R\&d_{i,t}+\lambda_{2}\,C\,o\,n\,t\,r\,o\,l\,s_{i,t}+\sum Y\,e\,a\,r+\sum I\,n\,d+\tau_{i,t}$$

(6) $$S\,u\,p\,\_\,R\&d_{i,t}=\gamma_{0}+\gamma_{1}\,C\,u\,s\,\_\,R\&d_{i,t}+\gamma_{2}\,A\,b\,i\,l\,i\,t\,y_{i,t}+\gamma_{3}\,C\,u\,s\,\_\,R\&d_{i,t}*A\,b\,i\,l\,i\,t\,y_{i,t}+\gamma_{4}\,C\,o\,n\,t\,r\,o\,l\,s_{i,t}+\sum Y\,e\,a\,r+\sum I\,n\,d+\theta_{i,t}$$

Following the prior literature (Kale and Shahrur, 2007; Shahrur and Raman, 2008), we used R&D expenditure to measure relationship-specific investment, that is, we created two variables: *Sup_R*&*d1*, which measures the R&D investment of suppliers, equaling R&D expenditure standardized by total assets, and *Sup_R*&*d2*, which is R&D expenditure scaled by operating income. Taking into account that a supplier firm may have multiple customers, we used the average R&D expenditure of customers to proxy the R&D investment of customer firms, *Cus_R*&*d1* and *Cus_R*&*d2*, calculated just as *Sup_R*&*d1* and *Sup_R*&*d2*.^7^

The regression results of models (5) and (6) are shown in Table 8. This shows that the coefficient of *Cus_R*&*d1* or *Cus_R*&*d2* is significantly positive (β = 0.2156, *t* = 2.81, *p* < 0.01; β = 0.1690, *t* = 2.40, *p* < 0.05), which indicates that the R&D investment of a supplier firm is indeed affected by customers. The interaction term of *Cus_R*&*d1* × *Ability* or *Cus_R*&*d2* × *Ability* is significantly negative (β = −0.5831, *t* = −2.39, *p* < 0.05; β = − 0.9806, *t* = − 2.45, *p* < 0.05), which indicates that managerial ability can significantly decrease the sensitivity of their R&D investment to customers. These results are consistent with the argument that high-ability managers always try hard to reduce their dependence on major customers, even when facing a larger listed company.

**TABLE 8:** The mechanism of the sensitivity of R&D investment to customers.

	(1)	(2)	(3)	(4)
	*Sup_R&d1*	*Sup_R&d1*	*Sup_R&d2*	*Sup_R&d2*
*Cus_R&d1*	0.2156***	0.1914**		
	(2.81)	(2.54)		
*Cus_R&d2*			0.1690**	0.1017
			(2.40)	(1.54)
*Ability*		−0.0194***		−0.0790***
		(−4.15)		(−5.46)
*Cus_R&d1* × *Ability*		−0.5831**		
		(−2.39)		
*Cus_R&d2* × *Ability*				−0.9806**
				(−2.45)
*Constant*	−0.0237	−0.0253	−0.0299	−0.0351
	(−1.00)	(−1.06)	(−0.58)	(−0.67)
Controls	Yes	Yes	Yes	Yes
Year FE	Yes	Yes	Yes	Yes
Ind FE	Yes	Yes	Yes	Yes
Observations	900	900	900	900
Adj. *R*^2^	0.414	0.434	0.397	0.455

We tested the sensitivity of R&D investment mechanism of managerial ability in this table, using a sample of 900 observations of the listed customer sample. The t-statistics are displayed in parentheses. The symbols ***, **, and * indicate significance at the 1, 5, and 10% levels, respectively.

## Conclusion

In this study, we examined whether and how managerial ability affects the relationship between customer concentration and corporate performance. The results show that customer concentration affects corporate performance negatively, but the managerial ability can mitigate this effect. Cross-sectional tests show that the negative effect of customer concentration is only significant in the subsample of low ability and lower efficiency in asset utilization, and the moderating effect of managerial ability is more significant for firms with lower gross margins, which indicates that managerial ability can reduce the value entrenchment effect of major customers through excellent operational ability. Additional analysis of mechanisms shows that competent managers select major customers who are more beneficial to their company and decrease the sensitivity of their R&D investment to customers, thus reducing their dependence on major customers and protecting corporate performance from extraction.

The study offers evidence indicating that manager heterogeneity plays an important role in company decision-making in the supplier–customer context, and managerial ability is critical to the enhancement of corporate performance when facing major customers. Although major customers are an important corporate resource, it is recommended for managers to reduce their dependence on major customers as much as possible because customer concentration has a negative impact on corporate performance. Besides superior operating ability, it is necessary to decrease customer-specific investment to make their products or services suitable to more customers. When selecting customers, it should concern the contribution to the company.

A limitation of this study is that we did not investigate the specific multifaceted nature of managerial ability at the manager level, while we focused on a comprehensive proxy of ability showing the efficiency in resource deployment at the firm level. Future studies may investigate the specific characteristics of managerial ability assisted by a cognitive or psychological methodology, further exploring whether the ability is matched at the supplier–customer level, and how the matching, if any, affects the relationship of customer concentration with corporate performance.

## Data Availability Statement

The original contributions presented in the study are included in the article/Supplementary Material, further inquiries can be directed to the corresponding author.

## Author Contributions

GJ conceived the presented idea, developed the theory, and verified the analytical methods. QJ and XL collected available data and performed the computation. All authors discussed the results and contributed to the final manuscript.

## Conflict of Interest

The authors declare that the research was conducted in the absence of any commercial or financial relationships that could be construed as a potential conflict of interest.

## Publisher’s Note

All claims expressed in this article are solely those of the authors and do not necessarily represent those of their affiliated organizations, or those of the publisher, the editors and the reviewers. Any product that may be evaluated in this article, or claim that may be made by its manufacturer, is not guaranteed or endorsed by the publisher.

## Footnotes

1 We divided the sample into five groups, and the coefficients of customer concentration becomes significantly positive in the subsample of the highest managerial ability, which further proved the moderating effect of managerial ability.

2 We also performed the same subsample regressions based on proxies of accounts receivable turnover (*Art*) and inventory turnover (*It*), and the results remained the same. The negative effect of customer concentration is only significant in the subsample of lower *Art* and lower *It*, and the coefficients are significantly different between the two groups, while the moderating effect is significant for both groups and the differences of the coefficients are insignificant between the subsamples.

3 We also adopted a 2-year lagged customer concentration, which is regularly used in solving the endogeneity issue caused by reverse causality, as an additional instrumental variable. Our results remained the same.

4 We constructed a dummy variable *CCdum* to measure *CC*, according to the average value of *CC* by industry and year for robustness testing, which equals 1 when bigger than the average value, and 0 otherwise. We used industry adjusted *Roe* (*iRoe*) as an alternative measure of corporate performance, calculated as *Roe* minus industry mean *Roe* by year. The results are all the same as our baseline results.

5 Excessive dispersion or concentration of corporate customers may be related to the strategic decision-making of the company, and it is difficult to distinguish the impact of managerial ability on the relationship between customer concentration and corporate performance. Thus, we excluded samples with a customer concentration of 1 and limited the sample to the largest customer sales rate greater than 10%. The results were not affected.

6
http://www.forbeschina.com

7 Only when a supplier firm discloses detailed information about its customers, and only if the customer firm is a listed company and also discloses its R&D expenditures, can we obtain the data required to calculate *Cus_R*&*d1* and *Cus_R*&*d2*, so the sample is much smaller in the regression of models (5) and (6) than the baseline regression.

## Appendix

**TABLE A1:** Definition of variables.

Variables	Definition
*Roe*	Net profit/shareholders’ equity at the end of the year
*CC*	Total sales amount of the top five customers/total sales amount of the year
*Ability*	Calculated using DEA–Tobit model developed by Demerjian et al. (2012)
*Size*	The natural logarithm of total assets at the end of year
*Grow*	The growth rate of operating income
*Lev*	Total debt/total assets
*First*	The share percentages held by the largest shareholder
*Age*	The natural logarithm of the firm’s listing year plus one
*Tangible*	Net fixed assets/total assets
*Indd*	The proportion of independent directors on board
*State*	A dummy variable that equals 1 if the firm is controlled by the state, 0 otherwise
*Board*	The natural logarithm of total number of directors
*Mb*	Total assets/market value
*Pay*	The natural logarithm of total monetary compensation
*Separation*	Proportion of ownership of actual controller/proportion of control rights
*Cf*	Net cash flow from operating activities/total assets at the end of year
*Ms*	The percentage of revenues earned by the firm within its industry
*Fcf*	A dummy variable that equals 1 if the company has positive free cash flow, 0 otherwise
*Bsc*	1−Σp_*i*_^2^, p_*i*_ is the proportion of industry i income in main business income
*Oos*	A dummy variable that equals 1 if the company has overseas subsidiaries, 0 otherwise
