# Supplementary material for: Customer Concentration, Managerial Ability, and Corporate Performance
Source: Front Psychol. 2022 Jan 17;12:814646. doi: 10.3389/fpsyg.2021.814646 (PMC8801520; doi:10.3389/fpsyg.2021.814646)
Supplement: Supplementary file 1 [file Table_1.DOCX]

***Supplementary Material***

**1 Supplementary Tables**

Table 1: Cross-sectional heterogeneity tests in *Art* and *It* of main effect

|  | (1) | (2) | (3) | (4) |
| --- | --- | --- | --- | --- |
|  | *Roe* | | *Roe* | |
|  | *Low Art* | *High Art* | *Low It* | *High It* |
| *CC* | -0.0134** | 0.0013 | -0.0266*** | -0.0011 |
|  | (-2.32) | (0.24) | (-4.71) | (-0.20) |
| *Size* | 0.0298*** | 0.0221*** | 0.0312*** | 0.0206*** |
|  | (16.35) | (15.33) | (18.72) | (13.21) |
| *Grow* | 0.0090*** | 0.0074*** | 0.0085*** | 0.0077*** |
|  | (8.03) | (8.11) | (9.72) | (6.30) |
| *Lev* | -0.1247*** | -0.1119*** | -0.1289*** | -0.1159*** |
|  | (-16.96) | (-16.78) | (-18.47) | (-16.39) |
| *First* | 0.0627*** | 0.0413*** | 0.0560*** | 0.0473*** |
|  | (6.76) | (5.34) | (6.58) | (5.65) |
| *Age* | -0.0277*** | -0.0108*** | -0.0227*** | -0.0155*** |
|  | (-14.72) | (-6.69) | (-12.95) | (-9.05) |
| *Tangible* | -0.1092*** | -0.1073*** | -0.0923*** | -0.1215*** |
|  | (-10.01) | (-14.09) | (-9.10) | (-14.74) |
| *Indd* | 0.0615*** | 0.0101 | 0.0345** | 0.0472*** |
|  | (4.28) | (0.75) | (2.57) | (3.26) |
| *State* | 0.0036 | -0.0058** | -0.0060** | 0.0049 |
|  | (1.11) | (-2.11) | (-2.04) | (1.62) |
| *Board* | 0.0133* | 0.0034 | 0.0175*** | 0.0062 |
|  | (1.89) | (0.56) | (2.63) | (0.95) |
| *Mb* | -0.0925*** | -0.0833*** | -0.1059*** | -0.0781*** |
|  | (-12.55) | (-13.02) | (-15.66) | (-11.07) |
| *Pay* | 0.0124*** | 0.0221*** | 0.0143*** | 0.0201*** |
|  | (5.46) | (11.99) | (6.92) | (9.89) |
| *Separation* | 0.0656*** | 0.0412*** | 0.0468*** | 0.0651*** |
|  | (3.85) | (2.84) | (2.93) | (4.21) |
| *Cf* | 0.3604*** | 0.4515*** | 0.3233*** | 0.5090*** |
|  | (17.71) | (28.75) | (19.02) | (27.73) |
| *Constant* | -0.7071*** | -0.6657*** | -0.7459*** | -0.6172*** |
|  | (-17.20) | (-22.33) | (-22.17) | (-17.61) |
| *Year FE* | Yes | Yes | Yes | Yes |
| *Ind FE* | Yes | Yes | Yes | Yes |
| *Observations* | 9977 | 9976 | 9977 | 9976 |
| *Adj. R^2^* | 0.180 | 0.254 | 0.222 | 0.216 |
| *P-values* | Chi2 (1) =3.00* | | Chi2 (1) =9.15*** | |

*Note*: We test the main effect between the cross-sectional variations in accounts receivable turnover (*Art*) and inventory turnover (*It*) in Table 1, “p-values” is used to test the coefficient equality between two subsamples. T-statistics are displayed in parentheses. ⁎⁎⁎, ⁎⁎, and ⁎ indicate significance at the 1%, 5%, and 10% levels, respectively.

Table 2: Cross-sectional heterogeneity tests in *Art* and *It* of moderating effect

|  | (1) | (2) | (3) | (4) |
| --- | --- | --- | --- | --- |
|  | *Roe* | | *Roe* | |
|  | *Low Art* | *High Art* | *Low It* | *High It* |
| *CC* | -0.0226*** | -0.0130** | -0.0280*** | -0.0140** |
|  | (-4.03) | (-2.27) | (-5.13) | (-2.51) |
| *Ability* | 0.2227*** | 0.1255*** | 0.1964*** | 0.1352*** |
|  | (24.52) | (16.75) | (26.26) | (16.25) |
| *CC*Ability* | 0.0992*** | 0.0440 | 0.1135*** | 0.1038*** |
|  | (2.95) | (1.40) | (3.79) | (3.06) |
| *Size* | 0.0262*** | 0.0219*** | 0.0292*** | 0.0196*** |
|  | (14.76) | (15.39) | (18.11) | (12.71) |
| *Grow* | 0.0087*** | 0.0070*** | 0.0079*** | 0.0082*** |
|  | (8.07) | (7.74) | (9.28) | (6.78) |
| *Lev* | -0.1263*** | -0.1158*** | -0.1261*** | -0.1195*** |
|  | (-17.75) | (-17.59) | (-18.68) | (-17.13) |
| *First* | 0.0541*** | 0.0384*** | 0.0477*** | 0.0455*** |
|  | (6.02) | (5.04) | (5.80) | (5.51) |
| *Age* | -0.0225*** | -0.0107*** | -0.0212*** | -0.0148*** |
|  | (-12.30) | (-6.77) | (-12.52) | (-8.75) |
| *Tangible* | -0.0474*** | -0.0885*** | -0.0290*** | -0.1112*** |
|  | (-4.39) | (-11.64) | (-2.87) | (-13.64) |
| *Indd* | 0.0628*** | 0.0069 | 0.0322** | 0.0471*** |
|  | (4.51) | (0.52) | (2.49) | (3.30) |
| *State* | 0.0069** | -0.0061** | -0.0038 | 0.0041 |
|  | (2.17) | (-2.28) | (-1.35) | (1.36) |
| *Board* | 0.0137** | 0.0060 | 0.0152** | 0.0109* |
|  | (2.01) | (0.99) | (2.37) | (1.70) |
| *Mb* | -0.0712*** | -0.0821*** | -0.0870*** | -0.0772*** |
|  | (-9.92) | (-13.01) | (-13.23) | (-11.10) |
| *Pay* | 0.0139*** | 0.0247*** | 0.0163*** | 0.0228*** |
|  | (6.29) | (13.55) | (8.18) | (11.31) |
| *Separation* | 0.0541*** | 0.0222 | 0.0286* | 0.0488*** |
|  | (3.28) | (1.55) | (1.85) | (3.19) |
| *Cf* | 0.3166*** | 0.4252*** | 0.2650*** | 0.4858*** |
|  | (16.02) | (27.32) | (15.95) | (26.76) |
| *Constant* | -0.6785*** | -0.7073*** | -0.7599*** | -0.6508*** |
|  | (-17.05) | (-23.88) | (-23.35) | (-18.76) |
| *Year FE* | Yes | Yes | Yes | Yes |
| *Ind FE* | Yes | Yes | Yes | Yes |
| *Observations* | 9977 | 9976 | 9977 | 9976 |
| *Adj. R^2^* | 0.233 | 0.274 | 0.273 | 0.237 |
| *P-values* | Chi2 (1) =1.02 | | Chi2 (1) =0.03 | |

*Note*: We test the moderating effect between the cross-sectional variations in accounts receivable turnover (*Art*) and inventory turnover (*It*) in Table 2, “p-values” is used to test the coefficient equality between two subsamples. T-statistics are displayed in parentheses. ⁎⁎⁎, ⁎⁎, and ⁎ indicate significance at the 1%, 5%, and 10% levels, respectively.

Table 3: Endogeneity test of additional instrumental variable

|  | (1) | (2) |
| --- | --- | --- |
|  | *Roe* | *Roe* |
| *CC* | -0.0199*** | -0.0306*** |
|  | (-3.23) | (-5.06) |
| *Ability* |  | 0.1757*** |
|  |  | (23.68) |
| *CC*Ability* |  | 0.2016*** |
|  |  | (5.75) |
| *Constant* | -0.7495*** | -0.7526*** |
|  | (-24.97) | (-25.60) |
| *Controls* | Yes | Yes |
| *Year FE* | Yes | Yes |
| *Ind FE* | Yes | Yes |
| *Under identification test* | Chi-sq (1) =3531.5*** | Chi-sq (1) =3586.654*** |
| *Weak identification test* | F statistic=2.9e+04 | F statistic=2.9e+04 |
| *Observations* | 16414 | 16414 |

*Note:* We adopt two-year-lagged customer concentration as instrumental variable in Table 3. “Under identification test” and “weak identification test” are used to test the validity of instrumental variables, we do not report R^2^ because it’s not of practical significance. T-statistics (Z-statistics) are displayed in parentheses. ⁎⁎⁎, ⁎⁎, and ⁎ indicate significance at the 1%, 5%, and 10% levels, respectively.

Table 4. Robustness tests

|  | **Dependent variable measured by i*Roe*** | | **Independent variable measured by *CCdum*** | | **Excluding samples with *CC* = 1** | | **limiting the samples to *CC1* > 10%** | |
| --- | --- | --- | --- | --- | --- | --- | --- | --- |
|  | *iRoe* | *iRoe* | *Roe* | *Roe* | *Roe* | *Roe* | *Roe* | *Roe* |
| *CC* | -0.0111** | -0.0204*** |  |  | -0.0105* | -0.0195*** | -0.0121* | -0.0259*** |
|  | (-2.07) | (-3.83) |  |  | (-1.95) | (-3.68) | (-1.79) | (-3.84) |
| *Ability* |  | 0.1662*** |  | 0.1481*** |  | 0.1660*** |  | 0.1702*** |
|  |  | (20.76) |  | (14.86) |  | (20.69) |  | (16.02) |
| *CC*Ability* |  | 0.1108*** |  |  |  | 0.1130*** |  | 0.1165*** |
|  |  | (3.30) |  |  |  | (3.51) |  | (2.80) |
| *CCdum* |  |  | -0.0041** | -0.0054*** |  |  |  |  |
|  |  |  | (-1.99) | (-2.65) |  |  |  |  |
| *CCdum*Ability* |  |  |  | 0.0412*** |  |  |  |  |
|  |  |  |  | (2.90) |  |  |  |  |
| *Constant* | -0.7322*** | -0.7479*** | -0.6927*** | -0.7167*** | -0.6859*** | -0.7018*** | -0.6120*** | -0.6238*** |
|  | (-21.72) | (-22.66) | (-20.38) | (-21.53) | (-20.34) | (-21.20) | (-14.68) | (-15.46) |
| *Controls* | Yes | Yes | Yes | Yes | Yes | Yes | Yes | Yes |
| *Year FE* | Yes | Yes | Yes | Yes | Yes | Yes | Yes | Yes |
| *Ind FE* | Yes | Yes | Yes | Yes | Yes | Yes | Yes | Yes |
| *Observations* | 19953 | 19953 | 19953 | 19953 | 19934 | 19934 | 11415 | 11415 |
| *Adj. R^2^* | 0.198 | 0.235 | 0.212 | 0.248 | 0.213 | 0.249 | 0.194 | 0.237 |

*Note*: We perform robustness tests in Table 4, including replacing the dependent variable with *iRoe*, replacing the independent variable with *CCdum*, excluding samples with a customer concentration of 1 and limiting the sample to the largest customer sales rate greater than 10%. T-statistics are displayed in parentheses. ⁎⁎⁎, ⁎⁎, and ⁎ indicate significance at the 1%, 5%, and 10% levels, respectively.
